# The Relationship Between Intensity Indicators in Small-Sided Soccer Games

**DOI:** 10.1515/hukin-2015-0040

**Published:** 2015-07-10

**Authors:** Casamichana David, Castellano Julen

**Affiliations:** 1 Department of Health and Food, European University of the Atlantic, Santander, Spain.; 2 Faculty of Physical Activity and Sport Sciences, University of the Basque Country (UPV/EHU). Vitoria-Gasteiz, Spain.

**Keywords:** specific task, football association, global positioning system, rate of perceived exertion, heart rate

## Abstract

The aim of the present study was to examine the relationship between different kinds of intensity indicators in small-sided soccer games. This descriptive correlational study included 14 semi-professional male soccer players (21.3 ± 2.3 years, 174 ± 4.0 cm, 73.4 ± 5.1 kg) from the same team. The players were monitored by means of heart rate monitors and GPS devices during 27 small-sided games of nine different formats, yielding a total of 217 recordings. After each game the Borg scale was used to give a rate of perceived exertion (RPE). The internal load indicators were the mean heart rate relative to the individual maximum (%HRmean) and the RPE, while those for the external load were the player load, total distance covered, distance covered in two intensity ranges (>18 km·h-1 and >21 km·h-1), and frequency of effort (in the same two intensity ranges). There was a significant moderate correlation (r=0.506) between the two internal load measurements (%HRmean and RPE). Although there were significant correlations of different degrees between various external load measurements, only the player load was significantly correlated with the internal load indicators (r=0.331 with %HRmean and r=0.218 with RPE). During training programes of this kind, it is necessary to consider a range of intensity indicators so as to obtain complementary information. This will enable coaches to more accurately assess the load imposed on players and therefore optimize the training process.

## Introduction

Quantifying the load imposed by coaching drills is a key aspect in terms of understanding how training can be used to optimize the performance of athletes (Mujika, 2013). Once a training schedule has been planned and prescribed, it is essential to determine what an athlete has actually achieved (Borresen and Lambert, 2009) or in the event that real-time information is available, how he or she is performing (Aughey and Fallon, 2010). This information about the training load (TL) can then be used by coaches not only to improve performance (Coutts et al., 2009), but also to avoid injuries and overtraining (Gabbett and Jenkins, 2011).

One way of assessing the criterion validity of the supposed internal TL indicators is to compare them with variables representing the external (or imposed) TL. This assumes that the training workload is a reflection of the external TL imposed by the coach, the hypothesis being that there is a cause-effect relationship between the two (Impellizzeri et al., 2005). In soccer, and particularly in small-sided games (SSG), the individual response (internal TL) to a given imposed training programe (external TL) may differ among players and, consequently, the quantification of individual training may be problematic (Flanagan and Merrick, 2002).

The methods used to quantify the internal TL include the rating of perceived exertion (RPE) and heart rate (HR) responses to training (Borresen and Lambert, 2009). More recently, the validity of what is called the session-RPE (sRPE) has also been examined by comparing it with established HR-based methods for evaluating the TL (Alexiou and Coutts, 2008; Impellizzeri et al., 2004) which have been correlated with other indicators of the internal and external TL in full sessions (Casamichana et al., 2013; Scott et al., 2013a; Scott et al., 2013b). Despite the practical interest provided by these studies, conclusive findings in regard to the criterion validity of the sRPE method have yet to be reported in relation to SSG, a training activity that is now widely used in different team sports (Ford et al., 2010) and which has been the object of numerous studies (Aguiar et al., 2012; Hill-Haas et al., 2011). When using SSG, coaches can modify pitch size, the number of players and/or the rules of the game in an attempt to achieve certain objectives, whether technical, tactical or physical (Aguiar et al., 2012; Casamichana et al., 2014; Hill-Haas et al., 2011). These different kinds of objectives may also be sought concurrently (Flanagan and Merrick, 2002; Köklü, 2012).

Research into these kinds of training activity has, to date, only studied the relationship between different intensity measurements related to the internal load. For example, studies have examined the relationship between the RPE and both blood lactate concentration and the heart rate (Coutts et al., 2009; Little and Williams, 2007) obtaining coefficients of r=0.60 with the heart rate and r=0.63 with blood lactate. This suggests that HR-based methods may be a relatively poor way of evaluating very high intensity (and/or short duration) exercises such as SSG. Consequently, measures of the internal TL might not be the best indicators of intensity in soccer, and it may be necessary to complement them with external indicators.

As a result of technological developments it is now much easier to automatically evaluate the external TL of several players at the same time. Furthermore, the use of GPS technology can quickly provide an objective assessment of the external load during specific kinds of soccer training (Castellano et al., 2011), thereby overcoming the traditional problem of lack of control over the actual training content (Flanagan and Merrick, 2002). A further point to note with respect to soccer is that it is characterized by intermittent efforts of maximal and sub-maximal intensity, and it would therefore seem useful, when evaluating the external TL, to consider variables related to this high-intensity activity.

To the best of our knowledge, no studies have yet examined the relationship between intensity indicators associated with the internal and external TL, in specific relation to soccer training. In an attempt to fill this gap, the present study sought to determine the correlations between these two kinds of indicators in the context of SSG, and therefore, to assess their validity as measurements of training intensity. Moreover, the relationship study was carried out in different SSG formats (with different numbers of players per team). The working hypothesis was that there would be a weak relationship between internal and external intensity indicators, especially for those variables related to high-intensity efforts.

## Material and Methods

### Sample

The participants were 14 semi-professional male soccer players (age: 21.3 ± 2.3; body height: 174 ± 4.0 cm; body mass: 73.4 ± 5.1 kg; Yo-Yo intermittent recovery test level 1, YYIRT1: 2384.6 ± 348.5 m) who played for the same team (senior division) at the regional level. They had played federation soccer for an average of 12.5 years prior to the study. Their standard training involved 3–4 sessions per week (each lasting around 90 min), in addition to one competitive match. All the players were notified of the research design and its requirements along with the potential benefits and risks, and all of them provided formal consent prior to the start. The Ethics Committee of the University of the Basque Country also gave its institutional approval of the study.

### Procedure

The internal intensity indicators used were the mean heart rate with respect to the individual maximum (%HR_mean_) and RPE. Despite the practical implications of these indicators, no study has yet verified their sensitivity in tracking variations in external load indicators in SSGs. Therefore, it is not clear to what extent these internal indicators may be associated with the actual work load produced as a result of SSG-based training. In the present study, the players’ training activities were monitored using GPS technology and the resulting activity categories were assumed to be constructs representing individual external indicators of intensity.

The study was based on a descriptive correlational design, and was conducted during the 2009–10 competitive season. In order to determine their individual maximal HR (HR_max_) all the players completed the YYIRT1 before beginning the study. This testing was carried out on an outdoor artificial pitch with the players wearing football footwear. The validity and reliability of this test has been reported elsewhere (Bangsbo, 2003; Krustrup et al., 2003). Training sessions were then held over an eight-week period on an outdoor artificial grass pitch and at similar times of day (20:00) in order to avoid any effect of circadian rhythms on the measured variables (Drust et al., 2005). Prior to data collection the players were familiarized with both the types of SSG and the material that would be used in training. Each session began with a 15-min standard warm-up (running, stretching and technical drills with the ball), followed by three different kinds of SSG lasting 6 min each (Castellano et al., 2013) interspaced with 5 min of passive recovery. The nine formats were as follows: possession play, regulation goals and goalkeepers and small goals (2 m wide × 1.2 m high) but no goalkeepers with a different number of players per team (3 vs. 3, 5 vs. 5, and 7 vs. 7). The pitch size was varied so as to maintain the relative area per player (≈210 m^2^), with a constant length:width ratio (the 3 vs. 3 on a pitch measuring 43 × 30 m, the 5 vs. 5 on a 55 × 38 m pitch and 7 vs. 7 on a pitch measuring 64 × 46 m). These nine SSG formats were each played three times (27 in total with 217 individual records) and were chosen as they are commonly used by soccer teams for training purposes. During rest periods, players were allowed to drink fluids *ad libitum*. All participants were advised to maintain their normal diet, with special emphasis being placed on a high intake of water and carbohydrates. The order of play was established in advance by random selection. Throughout the SSG, a high level of motivation across the different sessions was maintained by encouragement from coaches (Rampinini et al., 2007). In addition, eight balls were distributed around the edge of the pitch so as to maximize effective playing time.

Data for the two internal indicators of intensity was gathered as follows: the HR intensity was assessed using a short-range telemetry system (Polar Team System, Polar Electro Oy, Finland); Having already measured the HRmax by means of the YYIRT1, the mean heart rate (HRmean) obtained during training could then be expressed as a proportion of the individual maximum (%HRmean).

The RPE was obtained using the modified 10-point Borg scale (Foster, 1998) during the rest period between SSGs. As in previous studies (McGuigan et al., 2004) the players were asked to respond to a standard question: *“How was it, how do you feel about the workout?”*. This modified 10-point scale has been previously validated in the context of SSG (Coutts et al., 2009), and all participating players were familiar with its use prior to commencement of this study.

The players’ external TL was monitored and evaluated by means of portable GPS devices (MinimaxX v.4.0, Catapult Innovations) operating at a sampling frequency of 10 Hz and incorporating a 100 Hz triaxial accelerometer. The device was fitted to the upper back of each player using a special harness. The GPS devices were activated 15 min before the start of each training session, in accordance with the manufacturer’s instructions. The recorded data was subsequently downloaded to a PC and analysed using the software package Logan Plus v.4.4 (Catapult Innovations, 2010). The reliability and validity of the devices used in this study have been reported elsewhere (Castellano et al., 2011). The mean (±SD) number of satellites during data collection was 12.5 (±0.6).

The external TL indicators were as follows: (a) TD, total distance covered (m·min^−1^); (b) DHS, distance covered at high speed without limit (>18 km·h^−1^), in m·min^−1^; (c) DSS, distance covered at sprint speed without limit (>21 km·h^−1^), in m·min^−1^; (d) FHS, frequency of effort at high speed without limit (>18 km·h^−1^); and (e) FSS, frequency of effort at sprint speed without limit (>21 km·h^−1^) in n·min^−1^.

A further indicator used was the player load (PL), calculated using the data obtained via the triaxial accelerometer incorporated within the GPS device (Boyd et al., 2011; Casamichana et al., 2013; Gastin et al., 2013). The PL has shown high reliability, both within and between devices, thereby suggesting that accelerometers are a viable tool for tracking activity changes during exercise (Akenhead et al., 2013; Varley et al., 2011). The PL was calculated using the following formula and expressed as arbitrary units per minute of practice (AU·min^−1^):

$$\mathrm{Player-load}=\surd \left(\left({\left({\mathrm{aca}}_{\text{t}=\text{i}+1}-{\mathrm{aca}}_{\text{t}=1}\right)}^{2}+{\left({\mathrm{act}}_{\text{t}=\text{i}+1}-{\mathrm{act}}_{\text{t}=1}\right)}^{2}+{\left({\mathrm{acv}}_{\text{t}=\text{i}+1}-{\mathrm{acv}}_{\text{t}=1}\right)}^{2}\right)/100\right)$$

where *aca* is the acceleration in the anteroposterior or horizontal axis, *act* is the acceleration in the transverse or lateral axis, *acv* is the acceleration in the vertical axis, *i* is the current time, and *t* is time.

### Statistical analysis

The data is presented as means and standard deviations (±SD). The normality and homogeneity of variances were examined with Kolmogorov-Smirnov’s (*p*=0.20) and Levene’s tests (*p*>0.39), respectively. The relationship between the various internal and external intensity indicators was assessed using the Pearson’s correlation coefficient. The magnitude of correlation coefficients was considered as trivial (*r*<0.1), small (0.1<*r*<0.3), moderate (0.3<*r*<0.5), large (0.5<*r*<0.7), very large (0.7<*r*<0.9), almost perfect (*r*>0.9) or perfect (*r*=1; Hopkins, 2002). All statistical analyses were performed using SPSS v16.0 (SPSS Inc., Chicago Illinois, USA). Statistical significance was set at *p*<0.01.

## Results

**Figure 1:**
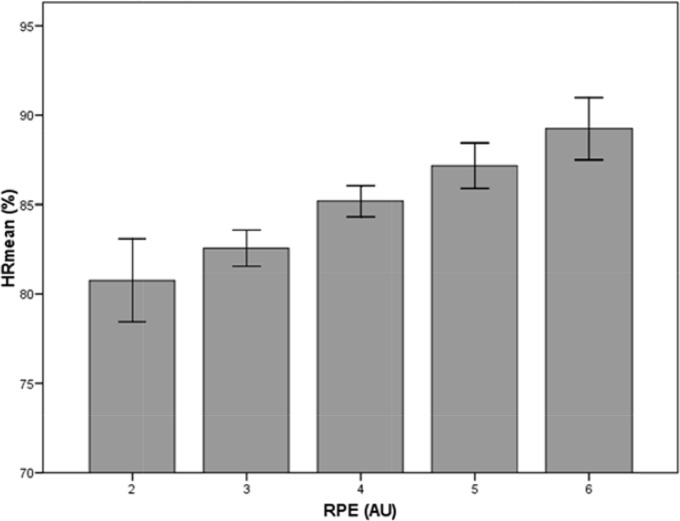
The mean heart rate (%) corresponding to each point score on the scale used to measure the RPE (n = 217). Bar heights are mean values and error bars are SD values.

The mean values obtained for the intensity indicators associated with the internal load were 84.6% (±4.3) for %HRmean and 3.8 AU (±1.0) for the RPE. The mean TD during the SSG was 118.3 m·min-1 (±18.7), while the mean PL was 15.8 AU·min-1 (±2.7). In terms of the distance covered in the two intensity ranges, mean DHS was 5.5 m·min-1 (±4.7) and mean DSS was 1.5 m·min-1 (±2.4). The two indicators of the internal load (%HRmean and RPE) were strongly correlated with one another (r=0.506, p<0.001; Figure 1).

**Figure 2:**
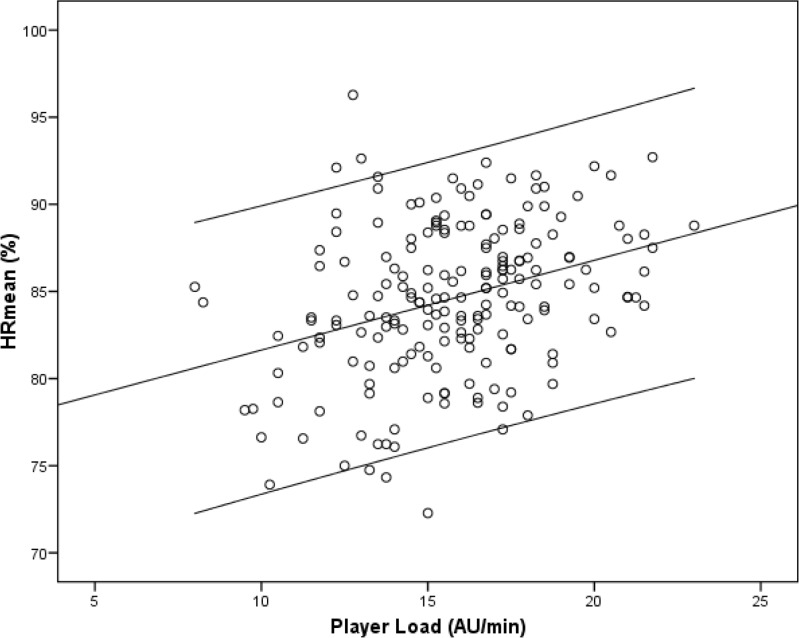
The relationship between the player load (by minute) and the HRmean (%) indicator (r=0.331, p<0.001). “AU” is the arbitrary unit.

There were no significant correlations between the RPE and the different external load indicators studied, except for a small correlation with the PL (r=0.218, p<0.001). As for the %HRmean, this measure showed a moderate correlation with the PL (r=0.331, p<0.001; Figure 2) and a small correlation with TD (r=0.258, p<0.001), there being no significant associations with any of the other indicators (DHS, DSS, FHS and FSS).

**Figure 3:**
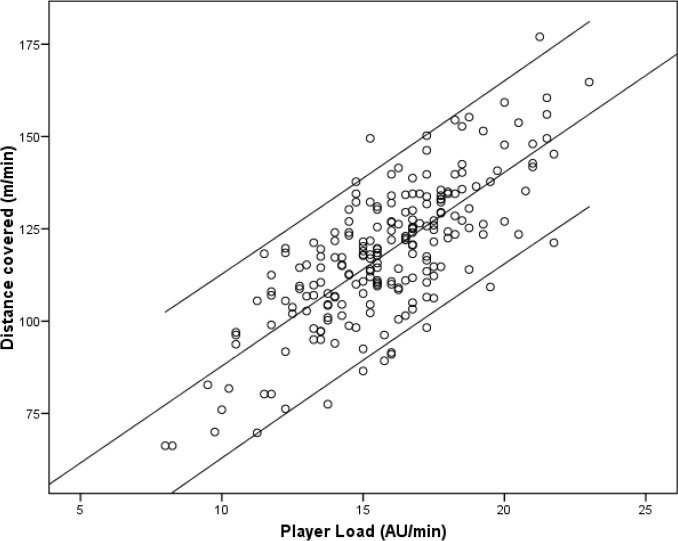
The relationship between the player load and TD (r=0.751, p<0.001), both assessed per minute. “AU” is the arbitrary unit.

The PL was strongly correlated with TD (r=0.751, p<0.001; Figure 3), but showed no significant correlations with DHS, DSS, FHS or FSS.

The different measurements used to assess high-intensity effort were very strongly correlated with one another, the effect sizes being either very large or almost perfect. To be more specific, DSS showed a very strong correlation with DHS (r=0.73, p<0.001), FHS (r=0.742, p<0.001) and FSS (r=0.894, p<0.001). DHS was also very strongly correlated with FHS (r=0.742) and FSS (r=0.701, p<0.001), while the association between FHS and FSS was almost perfect (r=0.907, p<0.001). In contrast to these results, TD was only significantly correlated with two of these high-intensity effort indicators, there being a small correlation with DSS (r=0.143, p<0.001) and a moderate one with DHS (r=0.327, p<0.001).

The relationship between different indicators in small-sided games of different formats (different number of players) is shown in Table 1. In general, we can observe that the magnitude of correlation is stronger in the 5-aside format (Table 1).

## Discussion

The purpose of this study was to examine the relationship between different internal and external load indicators in the context of SSG. The main conclusion to be drawn from the results is that in training programmes of this kind it is necessary to consider a range of intensity indicators so as to obtain complementary information and enable coaches to assess more accurately the load imposed on players. This is because in the majority of cases there is only a low or moderate correlation between various external load indicators, as well as between these and the internal load indicators.

Heart rate monitoring is a common practice in team sport games (Dellal et al., 2012). However, few studies have examined the relationship between this measurement of intensity and other intensity indicators associated with the external load, and the focus has always been on complete training sessions (Casamichana et al., 2013; Scott et al., 2013a; Scott et al., 2013b). These studies have reported very strong correlations between the HR and measurements of the external load such as the total distance covered or player load (Casamichana et al., 2013; Scott, Lockie et al., 2013), although the relationship is weaker between the HR and variables associated with high-intensity effort (Casamichana et al., 2013; Scott, Lockie et al., 2013). Given that the latter involve running intensities that exceed the individual maximal HR, monitoring the players’ HR may not be the most suitable indicator in these cases.

The results of this study suggest that the %HRmean is an intensity indicator that differs from the rest of variables, since the magnitude of its correlation with the various external load indicators was either moderate or small (with regard to the PL and TD, respectively), or null with respect to the distance covered and frequency of effort made at high or sprint speed (i.e. DHS, DSS, FHS and FSS). Given these results it would appear to be advisable to complement HR monitoring with another external load indicator present in high-intensity activity.

The RPE was strongly correlated with the %HRmean, but only weakly with the PL. Furthermore, the RPE showed no significant correlation with any of the other intensity indicators associated with the external load (i.e. TD, DHS, DSS, FHS and FSS). Significant correlations between the RPE and the %HRmean have been observed in previous studies of complete training sessions (Alexiou and Coutts, 2008; Borresen and Lambert, 2009; Clarke et al., 2013) most of which show moderate or high correlations. However, if one focuses on efforts of maximal or sub-maximal intensity then the relationship between the RPE and external load indicators appears less clear. For example, Casamichana et al. (2013) monitored complete training sessions in soccer and found significant correlations between the RPE and most of the external load indicators studied. However, strength of the correlation with indicators of high-intensity effort was at best moderate and at worst trivial. Scott et al. (2013b) also monitored soccer training sessions and found significant correlations between the session-RPE and all the external load indicators they used (r=0.43–0.84). Once again, however, the relationship became weaker when indicators of high-intensity activity were considered. An interesting observation was made by Scott et al. (2013a) in their study of Australian football. They found that although the session-RPE was strongly correlated with external load indicators, the magnitude of the relationship between the RPE (as a measure of intensity) and external indicators as a measure of intensity was only small or moderate.

Some previous studies, such as that by Coutts et al. (2009), have examined the relationship between load indicators in SSG rather than in complete training, and have reported similar results to the present study. The findings appear to support the idea that the RPE provides a global measurement of exercise intensity, one that does seem to be unduly affected by the presence of high-intensity effort (Casamichana et al., 2013). However, during SSGs, which are characterized by intermittent activity on the players’ part, the RPE may not be sensitive enough to detect small changes in high-intensity effort (Scott et al., 2013a).

Finally, it should be noted that the different variables studied here which were associated with high-intensity effort (DHS, DSS, FHS and FSS) were only correlated with one another, and as long as DHS and DSS were concerned, also with TD. This suggests that when monitoring intensity in activities such as SSG the assessment of the external load should consider one of these variables.

In relation to the study of relationships between variables in different SSG formats, we concluded that there are no clear differences regarding the number of players participating, which highlights the importance of respecting the above indications independently of the SSG format to be used in training. It is important to include the high-intensity effort assessment in order to know the external training load.

The information obtained from indicators associated with high-intensity activity could be of interest, particularly when the aim is to assess specific training drills such as SSG rather than just training sessions as a whole. Although there are numerous methods for evaluating the load imposed on players during training, coaches frequently rely on the HR and RPE, probably because these indicators have long been used for monitoring purposes in individual sports. However, they may not be sufficient when it comes to team sports, especially soccer. Therefore, during training programmes of this kind, it is necessary to consider a range of intensity indicators in order to get complementary information combining both internal and external indicators. In this way a more accurate measurement of the training load experienced by players will be obtained. This will enable coaches to assess the load imposed on players more accurately, and consequently optimize the training process, avoiding excessive or insufficient stimulation, which can lead, respectively, to overtraining or a lower level of fitness. By doing so, they can also help to reduce the likelihood of players developing unnecessary injuries.

## Figures and Tables

**Table 1:** Relationships between different intensity indicators in different SSG formats

SSG	Variable	RPE	%HR_mean_	TD	PL	DSS	DHS	FSS
7:7	%HR_mean_	.449^**^						
	TD	.237^*^	.267^*^					
	PL	.184	.138	.836^**^				
	DSS	.098	.13	.081	.041			
	DHS	.125	.208	.320^**^	.235^*^	.741^**^		
	FSS	.065	.101	.053	.049	.903^**^	.729^**^	
	FHS	.076	.083	.099	.073	.799^**^	.766^**^	.908^**^

5:5	%HR_mean_	.601^**^						
	TD	.371^**^	.597^**^					
	PL	.444^**^	.652^**^	.819^**^				
	DSS	−.094	−.028	−.163	−.115			
	DHS	−.073	.134	.021	−.016	.699^**^		
	FSS	−.045	.042	−.162	−.104	.906^**^	.684^**^	
	FHS	−.008	.129	−.076	−.072	.832^**^	.741^**^	.909^**^

3:3	%HR_mean_	.381^**^						
	TD	.194	.373^**^					
	PL	.053	.361^**^	.783^**^				
	DSS	.236	−.132	.109	.056			
	DHS	.129	−.032	.295^*^	.107	.565^**^		
	FSS	.169	−.143	.087	.020	.814^**^	.457^**^	
	FHS	.225	−.167	.051	−.036	.898^**^	.571^**^	.868^**^

RPE is the rate of perceived exertion; %HR_mean_ is the heart rate with respect to the individual maximum; TD is total distance covered; PL is the Player Load; DHS is distance covered >18 km·h^−1^; DSS is distance covered >21 km·h^−1^; FHS is frequency of effort at >18.0 km·h^−1^ and FSS is frequency of effort at >21.0 km·h^−1^, all variables assessed per minute.
